# Effect of Li, Na and K Modification of Alumina on its Physical and Chemical Properties and Water Adsorption Ability

**DOI:** 10.3390/ma12244212

**Published:** 2019-12-15

**Authors:** Sergey Reshetnikov, Irina Kurzina, Alesia Livanova, Eugene Meshcheryakov, Lyubov Isupova

**Affiliations:** 1 Boreskov Institute of Catalysis, Siberian Branch, Russian Academy of Sciences, Novosibirsk 630090, Russia; reshet@catalysis.ru (S.R.); isupova@catalysis.ru (L.I.); 2 Faculty of Chemistry, National Research Tomsk State University, Tomsk 634050, Russia; truelivanova@mail.ru (A.L.); meevgeni@mail.ru (E.M.)

**Keywords:** aluminium oxide material, material modification with ions, water adsorption, dynamic adsorption

## Abstract

The effect of alkali metal (Li, Na, K) incorporation on the morphology and water vapor uptake properties of mesoporous Al_2_O_3_ has been studied. The modification of the raw material, pseudoboehmite, represented a mixture of low-temperature phases (γ + η + χ)-Al_2_O_3_, and has been done at low-temperature that does not change the phase ratio. A decrease in specific surface values and an average pores size increase were observed as a result of the introduction of metal cations by impregnation and subsequent thermal treatment. The influence of the content of the modifying metal on the adsorption ability of the obtained samples in relation to water vapours has been studied. It has been established that alkaline modification Al_2_O_3_ with the lithium cations did not result in adsorption ability improvement, whereas samples that were modified with sodium or potassium in the amount of 1.2 weight % and 2.6 weight %, respectively, possess a higher equilibrium capacity (by ~40%), as compared to that of the initial sample (Al_2_O_3_), and a sufficiently high adsorption rate.

## 1. Introduction

Materials that are based on active aluminium-oxide are widely used as catalysts in the processes of oil refining (reforming, hydraulic cleaning, hydrocracking, etc.) and adsorbents, in particular, for air drying and natural gas dehydration [1,2]. Drying is necessary as a moisture presence at low temperature leads, for example, in the formation of hydrocarbons hydrates, which, accumulating in a gas pipeline, can cause their partial or complete occlusion, thereby disrupting the normal operating conditions of the gas main. The adsorption ability by water at different humidity for various sorts of aluminium oxide can significantly differ, but in all cases it increases with the increase in the gas humidity [3,4,5]. In this connection, the search for new, more efficient adsorbents is still relevant. The adsorbents efficiency can be increased by the purposeful changing of texture characteristics (specific surface and porous structure), phase composition, including the introduction of different modifiers influencing the number and power of the adsorption sites.

In works [6,7] research into the synthesis of highly-efficient aluminium oxide materials based on low-temperature forms of aluminium oxide (η-, γ- and χ-), as obtained by calcination of the products of alkaline hydration of thermally activated aluminium oxide containing 50% and more of the byerite phase, was conducted. A peculiarity of bayerite-containing hydroxide is the formation of low-temperature phases of aluminium oxide (primarily η-modification) at a calcination temperature of > 300 °C. This allows for obtaining samples with a large specific surface, with a required combination of surface sites, a larger number of micropores, and, respectively, with a greater static capacity than that of γ-Al_2_O_3_-based materials, which were obtained based on pseudoboehmite by reprecipitation.

The water vapour adsorption on active aluminium oxide is the combined result of three processes: chemosorption, physical adsorption, and capillary condensation. A monolayer of hydrogen bonded water molecules covers the surface hydroxyls, such that the H_2_O:OH ratio is 1:2 and this hydrogen bonded water is sometimes described as «quasi chemisorbed». An additional layer of water molecules might be physically absorbed on the top of the hydrogen bonded layer and, depending on the pore size and relative pressure, they may also be capillary condensation within the smaller (meso and macro) pores [8]. 

Chemical modification of the surface with acids and bases, leading to a change in the concentration of acid-base sites and reaction capacity of the aluminium oxide surface, is one of the methods of increasing adsorption ability of active aluminium oxide [9,10]. Thus, when introducing sulfuric acid at the stage of preparation of the formable mass, sorbents, which were obtained from pseudoboehmite-containing aluminium oxide, turned out to be comparable with boehmite-based adsorbents by the dynamic capacity, and they even surpassed them by the value of static capacity [3]. This was related to the change in the phase composition, texture characteristics, and acid-base properties of the surface. At the same time, the greatest modifying effect was observed for the γ-Al_2_O_3_-based materials, for which, after the introduction of sulfate ions, the number of acid hydroxyl sites and strong Lewis acid sites (LAS) turned out to be greater, and the average diameter of pores decreased. In work [11], aluminium oxide materials that were modified with sodium and modified with potassium in the amount of ~2 wt % were studied. It was established that alkaline modification of the aluminium oxide adsorbents surface led to an increase in the adsorption ability when compared to the initial, unmodified aluminium oxide. A correlation between equilibrium values of adsorption capacity of the studied samples and acid-base properties of the surface was observed.

Conducting a systematic study on identifying an interrelation among the concentration of the modifying alkaline metal, texture characteristics of adsorbents, in particular, based on aluminium oxide, and their adsorption ability in relation to water is relevant.

An increase in the effectiveness of porous materials–adsorbents is due to both the increase of their adsorption capacity in relation to water vapours and improvement of their dynamic characteristics–adsorption rate owing to the change in acid-base properties of the surface. Data on the dynamics (kinetics) of adsorption obtained on the fine fraction of the adsorbent, when pore-diffusion resistance is absent, which is typical of granules of the industrial size, are necessary, to specify the role of chemical modification in the change of adsorption characteristics.

The purpose of this work is the research on the establishment of the interrelation among the content of the modifying alkaline metal (Li, Na, K), texture characteristics of the γ-Al_2_O_3_-based materials, and their adsorption ability in relation to water.

## 2. Materials and Methods 

### 2.1. Synthesis of Adsorbents 

In this work, an aluminium oxide sample was obtained based on pseudoboehmite by centrifugal-thermal activation of hydrargillite (CTA HG) in the centrifugal flash-reactor TSEFLAR^TM^ (Novosibirsk, Russia) of the drum type at 700 °C [7] was used. The obtained sample was initially for the subsequent alkaline modification with potassium, sodium, and lithium ions. 

A method of impregnation from the solution excess (equilibrium deposition filtration) was chosen to modify adsorbent granules based on aluminium oxide (Al_2_O_3_) [12]. 300 ml of distilled water was poured into a one-liter glass tumbler, and the calculated amount of the corresponding alkali (NaOH or KOH or LiOH) was added and then mixed until its complete dissolution. Subsequently, the adsorbent granules of cylindrical shape (diameter is −3.55 ± 0.15 mm, height is 5.20 ± 0.80 mm (220 g) were dipped in this solution and held for 24 h while stirring, and they were then filtered and dried at 120 °C. After that, the samples were incinerated at 500 °C for 4 h in the muffle electric furnace.

### 2.2. Methods

The following methods were used to study the physical and chemical properties of the samples.

X-ray phase analysis (XRD) was made on the diffractometer Rigaku Miniflex 600 (Rigaku, Japan) with the following parameters of scanning: measurement range - 2θ = 10–90° with CuKα radiation, voltage = 40 kV, current = 15 mA, scanning = 2 grad·min^−1^. The diffractograms were decoded while using the ICDD database of PDF2 version.

Texture characteristics of the adsorbents were determined by the isotherms of nitrogen adsorption at 77 K while using the automatic gas-adsorption analyzer 3Flex (Micromeritics, USA). The specific surface was measured by the BET method. The mesopore volume was calculated, analyzing an integral curve of pore volume distribution, depending on their radius (by the adsorption branch); the average diameter of pores (in nm) was calculated according the formula d_av_ = 4000V_pores_/A, where A is the granule surface area [13]. 

The mass fraction of metals in the samples of aluminium oxide adsorbents was determined by atomic emission spectroscopy of microwave plasma with preliminary “exposure” of acids in the mixture in the system of microwave sample preparation on the device Agilent 4100 MP-AES (Agilent Technologies, Australia).

The weight method determined the adsorption amount [11] while using the spring quartz balance. The balance sensitivity was 2.9 × 10^−3^ g/mm. An optimal sample of the adsorbent was selected (0.02 ÷ 0.03 g), which allows for placing the 0.5–1.0 mm adsorbent fraction into the cup made of aluminium foil in one layer. Each sample was regenerated at a temperature of 200 °C in the argon flow before performing the adsorption tests (Ar of high purity containing not more than 10 ppm of admixtures) supplied at a rate of 5 L/h during an hour. The temperature of samples regeneration is 200 °C, all physically adsorbed water is removed, and partial dehydroxylation of the surface occurs.

Argon that was passed through two Drexel bottles, filled with distilled water (100% humidity), was supplied to the sample at 25 °C to carry out the process of water vapour adsorption. A series of experiments was made at different, subsequently increasing rates of flow to exclude the factor of influence of the rate of water vapours supply to the outer surface of the granules. According to the experimental results a gas flow rate, equal to 30 L/h, was selected.

## 3. Results

### 3.1. Material Characterization 

The prepared samples were modified with lithium, which contained 1.3, 2.0 and 4.1 wt % of the metal, with potassium (1.6, 2.0, 4.5, 5.4 wt %), sodium (1.2, 2.4, 4.0, 4.6 wt %), and the initial sample of the unmodified aluminium oxide Al_2_O_3_ were studied while using a complex of physical and chemical methods. According to the XRD results, the samples of aluminium oxide materials under study represented a mixture of low-temperature modifications of aluminium oxide—(γ + η + χ)-Al_2_O_3_. A ratio of phases during modification practically did not change. The samples that were modified with lithium and potassium contain sodium in impurity amounts, according to the results of atomic emission spectroscopy of microwave plasma, which is due to its presence in the initial samples (Table 1).

**Figure 1:**
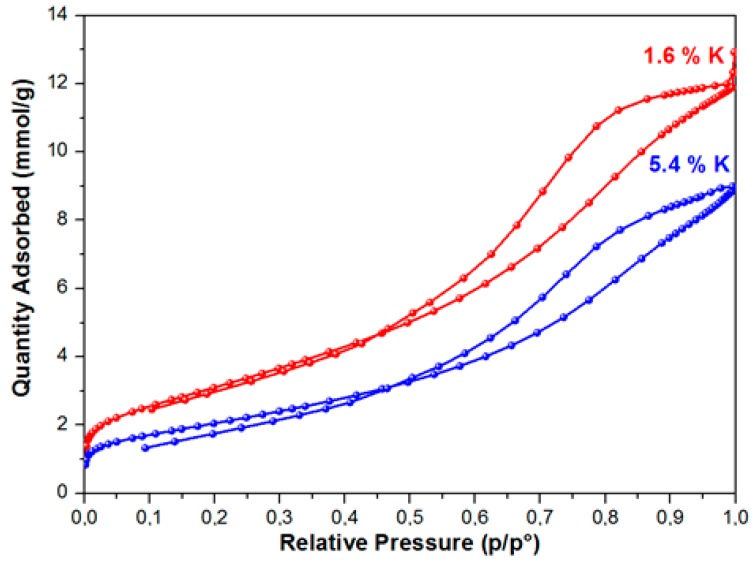
Isotherms of low-temperature adsorption-desorption of nitrogen on samples 1.6 K and 5.4 K.

As a result of studying the of nitrogen adsorption process, it was established that the BET equation described the isotherms of nitrogen adsorption at relative pressures of 0.05–0.3 on the samples. These isotherms belong to isotherms of IV type, according to the IUPAC classification with an initially sudden rise at a low relative pressure, which the presence of micropores and a gradual increase of the adsorption value with a pressure increase conditions (Figure 1). Figure 1 shows the nitrogen adsorption isotherms for samples with a high and low specific surface area.

**Figure 2:**
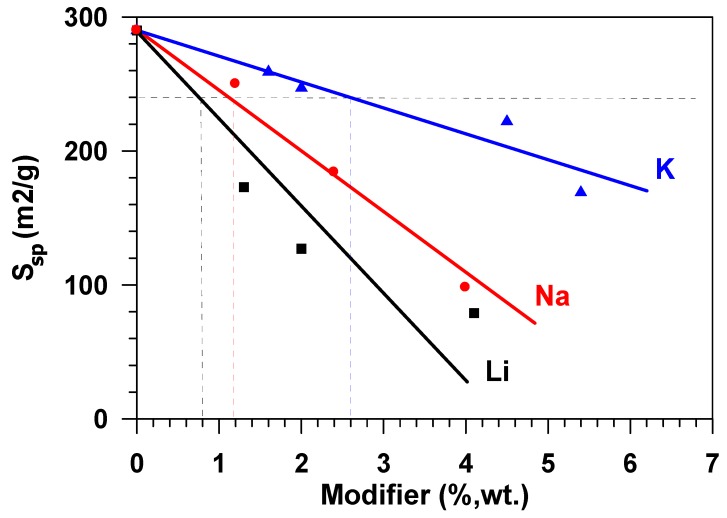
Effect of the Al_2_O_3_ modification with Li, Na, K on the specific material surface (S_sp_).

In the range of relative pressures over 40 %, the adsorption-desorption isotherms exhibited pronounced hysteresis. This type of the isotherm points to the presence of mesopores on the surface of the samples under study, as well as the presence of reversible capillary condensation in adsorbent mesopores. The following was determined for the samples under study while using the isotherms of low-temperature adsorption and desorption of nitrogen: specific surface area, porosity, and pore size distribution. During the modification of the aluminium oxide material with the atoms of alkaline metals (Li, Na, K), texture characteristics of the material change—along with the increase of the modifying metal content, the specific surface of the material and the total pore volume decrease. The greatest decrease of S_sp_, which was calculated while using the weight percent of metal content, was observed in the samples modified with lithium, the least, with potassium. Thus, for example, with the modifying metal content equal to 2.6 wt %, for samples that were modified with lithium, the surface of the initial Al_2_O_3_, amounting to 290 m^2^/g, approximately decreased by up to 120 m^2^/g, i.e., 2.4 times (Figure 2), whereas a decrease in the specific surface was ~40% and ~17%, respectively, for samples that were modified with sodium and potassium.

**Figure 3:**
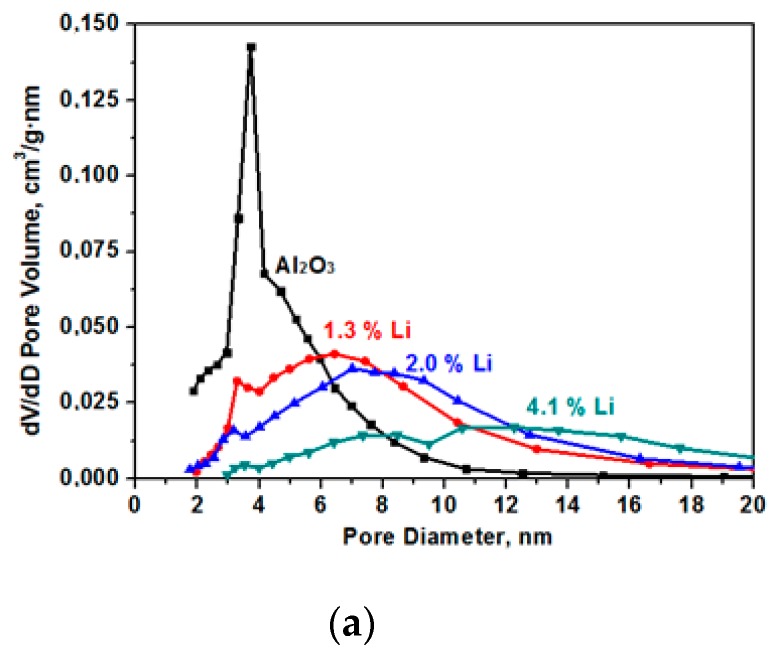

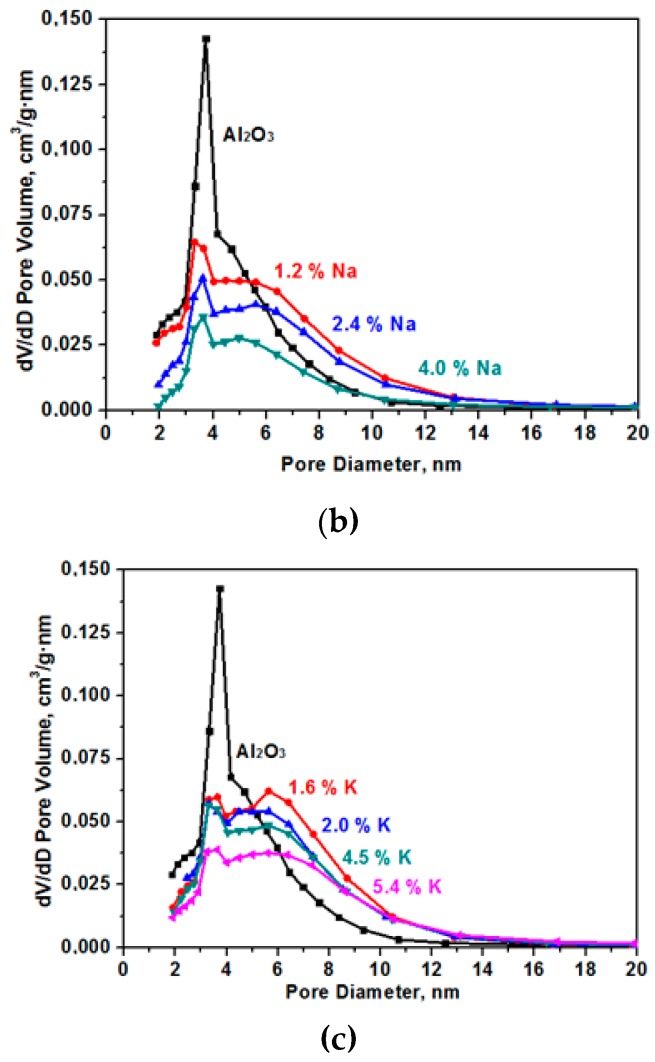
Pore size (*D*) distribution for the samples for the Al_2_O material modified with Li (**a**), Na (**b**), and K (**c**).

The specific surface decreases as a result of sintering in the presence of alkaline cations [14]. The data on pore size distribution in the samples that are presented in Figure 3 show the presence of only small mesopores in the samples. In the initial (Al_2_O_3_) sample, the average pore size was 4.7 nm with a strongly pronounced peak in the range of the pore diameter equal to 3.8 nm. As a result of samples modification, the number of fine pores decreases and the number of larger pores increases.

Thus, modifying the aluminium oxide samples with alkali metal ions, it is possible to control the specific surface and porous structure of the obtained materials.

### 3.2. Effect of Flow Rate on Water Vapour Adsorption

**Figure 4:**
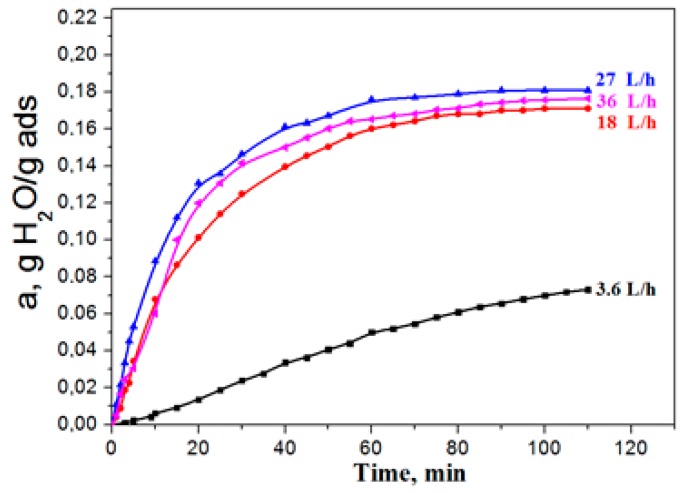
The influence of the gas flow rate on adsorption dynamics.

Aluminium oxide has become widely used as an adsorbent, in particular, for water vapours dehydration. The essence of adsorption dehydration consists in the selective adsorption by the pore surface of a solid material-adsorbent of water molecules with their subsequent extraction from pores by external influences (by increasing the adsorbent temperature or decreasing the medium pressure). Process behavior, when external diffusion resistance is absent, i.e., when water vapour supply to the grain surface is not limited, is the key factor of successful exploitation of adsorbents. When conducting the kinetic experiment, the adsorption dynamics was studied, depending on the delivery rate of the gas flow in a wide range of 3.6–36.0 liters per minute, to determine the gas delivery rate (volume) providing the absence of external diffusion. It was found that, at water-vapour flow rates of less than 25 l/min., the adsorption capacity grew with the increase of the gas delivery rate for each adsorption period (Figure 4).

The growth of the adsorption capacity, along with the increase in the flow rate, points to the fact that the process proceeds in the area of influence of external diffusion, i.e., the rate of water vapour delivery from the flow to the adsorbent surface limits the adsorption rate as a whole is limited (external diffusion to exterior surface of particle). With the increase in the flow rate, in the range of 25–35 L /min the adsorption capacity of the sample did not depend on the consumption, and it can be concluded that the adsorption process takes place in the kinetic mode. Therefore, all of the experiments presented further were made at a flow consumption of 30 L/h.

### 3.3. Dynamics of Water Adsorption Study

The adsorbent effectiveness is determined by not only static (equilibrium) capacity, but also by the adsorption rate. These two factors determine the time of protective action of the layer in the adsorber, which can provide a required degree of gas flow dehydration. Even if the adsorbent has a high static capacity, but a low adsorption rate, the shape of the adsorptive curve in the adsorbent layer will be flat; therefore, there will be a quick “breakthrough” of water, which is higher than the allowable concentration. On the contrary, in the case of a high adsorption rate, but low adsorbent capacity, the layer will quickly reach the maximum saturation, having not provided a high dynamic capacity of the layer. Consequently, effective adsorbents must possess high capacity and a high adsorption rate. Data on adsorption dynamics (kinetics) obtained on a fine fraction of the adsorbent when the pore-diffusion resistance is absent are necessary for specifying the role of chemical modification in the change of the adsorption characteristics.

**Figure 5:**
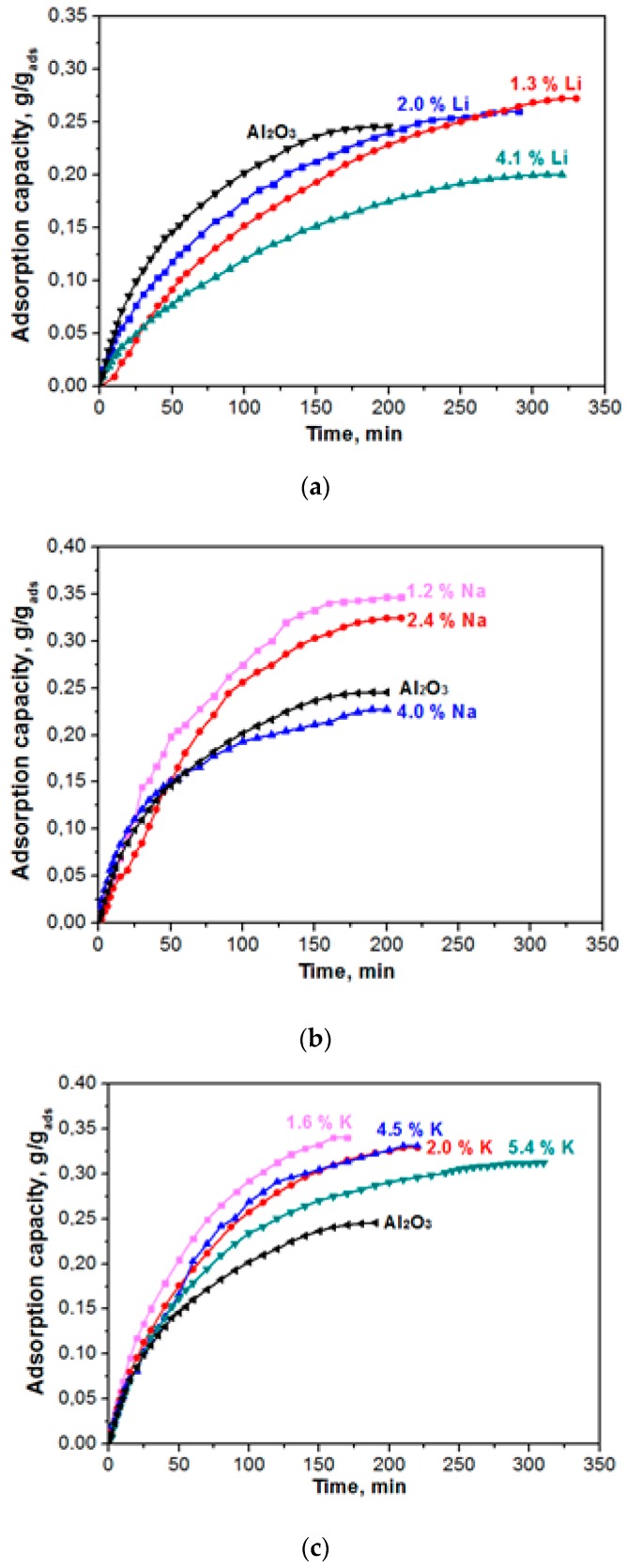
Kinetic (dynamic) curves of water vapour adsorption (0.5–1.0 mm fraction; flow rate of the gas being adsorbed, 30 L/h) for the Al_2_O_3_ material modified with Li (**a**), Na (**b**), and K (**c**).

Figure 5 shows the kinetic curves of water-vapour adsorption on the samples of adsorbents that were modified with alkaline metals (Li, Na, K). The kinetics of actual adsorption of water on the sites of alumina is very fast. However, a substantial resistance to mass transport can be exhibited by the finite diffusivity of water molecules from the external gas phase to the adsorption sites through the porous network of the adsorbent particle [15]. Mathematical modeling of the experimental data was carried out to quantitatively assess the adsorption rate value since the adsorbents capacity differs. A model that was proposed by Glueckauf [16] is widely used to model the dynamics of water vapours adsorption by the surface of hydrophilic adsorbents (zeolite, aluminium oxide, salts, etc.). Vapour adsorption in isothermal conditions occurs under constant partial pressure, and the adsorption process is described by the following differential equation:

da/dt = β(a* − a) (1)

with initial conditions:

*t = 0: a = 0.* (2)

where a* is adsorption value, equilibrium with the current concentration of the substance in the flow on the external surface of the granules; *a* is current value of the adsorbed substance; *β* is the kinetic coefficient, denoting the adsorption rate constant, min^−1^; and, *t* equals time, min. 

Glueckauf equation is often applied for describing the water vapour kinetics adsorption by the particles of active aluminium oxide [15]. Let us note that this equation is used when the internal diffusion limits the process, since it is an empirical approximation of the equation of internal diffusion kinetics describing substance diffusion in the pores of the adsorbent granule:

*da/dt = div(D_e_ grad C),*

where *D_e_*—effective diffusion coefficient, while taking the mass exchange in the grain into account; *C*—adsorbed substance concentration. 

After transformations, Equations (1) and (2) takes the following form:

(3) $$\frac{a}{a^{*}}=1-e^{-\beta \tau }$$

Equation (3) can be presented in a linear form:

−ln(a^*^ − a) = βτ − ln a^*^ (4)

It is possible to graphically calculate the coefficient β by the slope of the line while using the diagram of experimental dependence ln(a^*^ – *a*) on time. 

Experimental data on adsorption dynamics of the samples under study, as presented in Figure 5, were processed according to Equation (4). Table 2 gives the parameters of model (1) and (2) for samples differing by the content of alkaline metals (Li, Na, K). 

The initial aluminium oxide sample, whose specific surface is equal to 290 m^2^/g, has equilibrium adsorption capacity that is equal to 0.25 g of water per gram of the material, i.e., in the adsorbent there is 25% of water by weight. According to Taylor theory, substances with the heterogeneous surface interact (react) only in special areas, so-called active sites [17]. The number of atoms per unit of the metal surface to some degree depends on the crystallographic face, but for all of the metals it is approximately equal to 10^19^ atoms per square meter [18]. Consequently, having assumed that water molecules uniformly cover the inner surface of the material, then in this case ~ 3 molecules of H_2_O are adsorbed at one active site.

**Figure 6:**
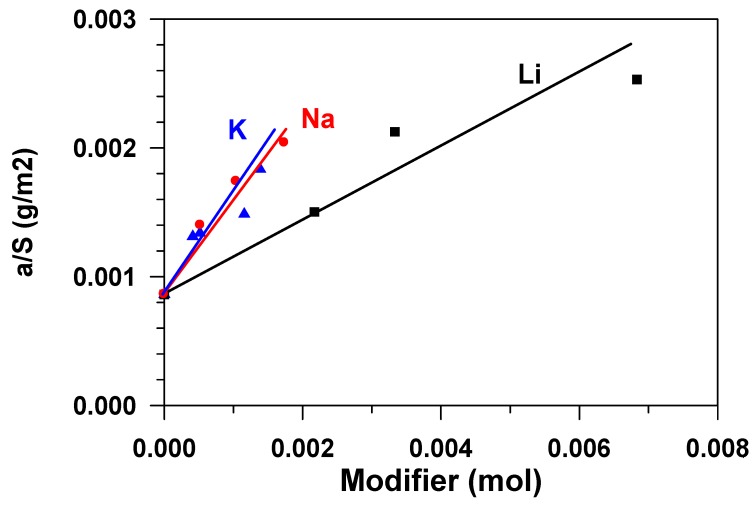
Dependence of the capacity of the adsorbent surface unit on the mole content of the modifier.

The chemical nature of the active sites that are responsible for adsorption phenomena on aluminas is still not well understood. Defect structures that formed via surface dehydroxylation result in localized regions of adsorptive and catalytic activity. For adsorption, active aluminas can be considered as possessing both Lewis and Bronsted acidic and basic sites of various strengths and concentrations. Acidity is contributed by lattice Al^3+^ ions, protonated hydroxyls, and some acidic hydroxyls. Basicity is a result of O^2−^ anion vacancies and basic hydroxyls [3]. The alkaline modification of aluminium oxide influences both texture characteristics–the specific surface and the porous structure of the material (Table 1), and adsorptive ability of the surface (S_sp_) in relation to water. Figure 6 presents a dependence of the equilibrium adsorbent capacity (a^*^), classified as the sample specific surface (Figure 2) on the amount of the modifier, expressed in the moles of the substance. The figure shows that the specific adsorption ability grows with the increase in the modifier content, which is apparently connected with the change in acid-base properties of the surface [7], namely, with the growth in the number of strong basic sites [10]. The fact that the total basicity of the surface of aluminum oxide increases with the increase in the content of alkali metals in it was previously noted in [3]. The modification with atoms (cations) of sodium and potassium exerts stronger influence on the specific adsorption ability, while that with lithium cations, was less.

**Figure 7:**
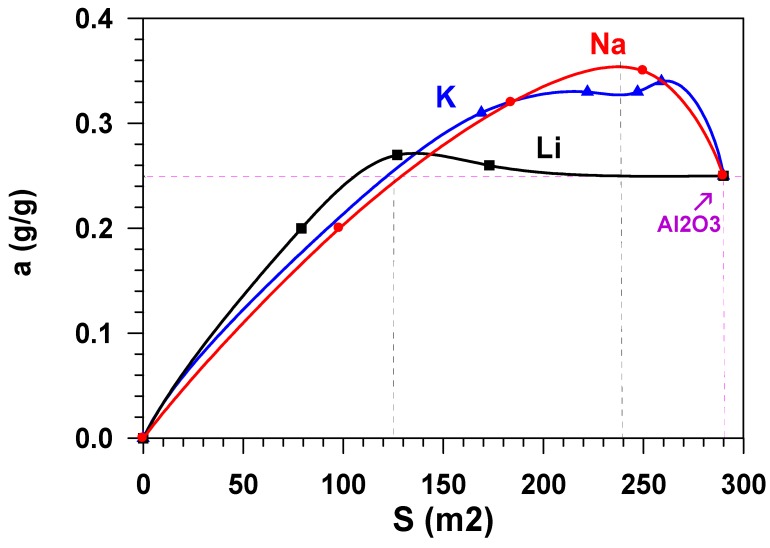
Dependence of the adsorption capacity of the modified materials (a^*^) on the specific surface (S_sp_).

The growth in the adsorption capacity of the samples, when the content of alkaline metals cations increases (Figure 6) is accompanied by the decrease in their specific surface. Figure 7 shows the dependence of the equilibrium adsorption capacity of the modified materials (a^*^) on the specific surface (S_sp_). When modifying Al_2_O_3_ with alkaline metals, the specific surface decreases (Figure 2), but the adsorption ability of the material surface unit increases. It is possible to note that modification with lithium does not practically influence the adsorption capacity. At the same time, the dependence of the capacity of samples, modified with potassium and sodium, on the specific surface is of extreme nature in the range of change S_sp_ = 125–290 m^2^/g. The maximum value of the equilibrium adsorption capacity is observed in the range of S_sp_ = 240 ± 24 m^2^/g. This value of the specific surface (shown in Figure 2 as a horizontal dotted line) is provided by modification of the initial material with 1.2 wt % of sodium or with 2.6 wt % of potassium (shown in Figure 2 as vertical dotted lines). While taking the molecular weight of sodium (23 g/mole) and potassium (39 g/mole) into account, this approximately amounts to an identical value that is equal to 5.2⋅10^−4^ and 6.8⋅10^−4^ moles per gram, respectively. When considering the fact that the number of atoms–active sites–in the monolayer (ML) is approximately equal to 10^19^ atoms per square meter [18,19], the number of active sites on the surface of Al_2_O_3_ modified with sodium is ~0.13ML, with potassium being 0.17 ML. Let us note that, at the same value of the specific surface (S_sp_ = 240 m^2^/g.), the weight content of lithium in the sample is 0.8 wt % (Figure 2), which amounts to 0.3 ML, i.e., 30 %.

The data in Figure 7 show that adsorptive capacity by water vapours of the obtained samples does not practically change when modifying aluminium oxide with lithium cations. However, alkaline modification of the aluminium oxide adsorbents surface with potassium cations or sodium cations results in the increase of the equilibrium adsorption capacity (a^*^) by ~40% as compared to the initial unmodified sample.

**Figure 8:**
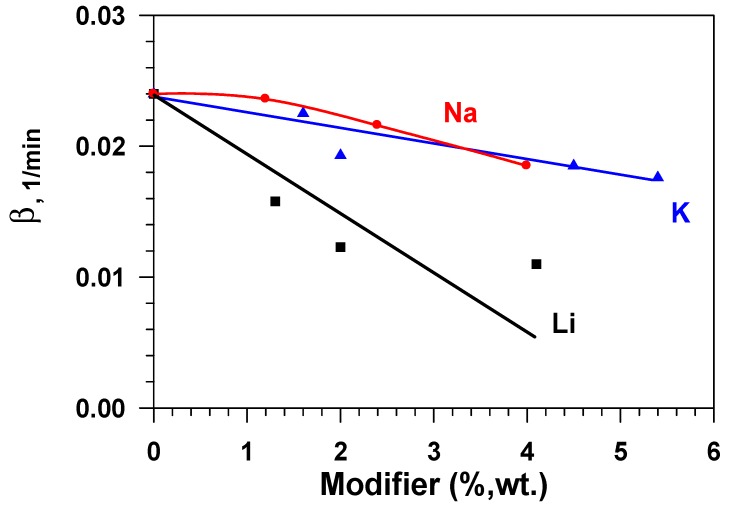
Dependence of the water adsorption rate constant (β) on the modifier concentration (Li, Na, K) in aluminium oxide.

Figure 8 presents dependences of the constant of the water adsorption rate (β) on the modifier concentration (Li, Na, K) in aluminium oxide while taking the fact that the effectiveness of the adsorbent layer is determined by not only static (equilibrium) capacity, but also the adsorption rate, into account. These data show that the adsorption rate constant that is calculated by Equation (4) decreases sharply with the growth in the modifier content for samples doped by lithium and, to a lesser degree, for samples containing sodium and potassium. Consequently, samples that are modified with sodium or potassium have a higher equilibrium capacity, as compared to the initial sample (Al_2_O_3_), and a quite high rate of adsorption in relation to water vapours.

## 4. Conclusion

In this work, the influence of the content of the modifying alkaline metal: lithium, sodium, and potassium (in the amount up to ~ 5.0 wt %) on the texture characteristics of materials, based on aluminium oxide obtained from pseudoboehmite-containing hydroxide by the technology of centrifugal-thermal activation, as well as on their adsorption ability in relation to water, has been studied. The following conclusions can be drawn:

(1) According to the XRD results, modification did not influence the change of the phase composition—all of the samples of the aluminium oxide material represented a mixture of low-temperature modifications of aluminium oxide—(γ + η + χ)-Al_2_O_3_. The presence of fine mesopores with an average diameter in the range of 4-15 nm was typical of all the obtained samples. As a result of the Al_2_O_3_ modification with alkaline metals (Li, Na, K), the values of the specific surface decrease from 290 m^2^/g and the average pore size increases.
(2) Al_2_O_3_ modification led to an increase in the equilibrium adsorption capacity of the materials mass unit in relation to water vapours. The Na and K cations exerted the most substantial influence.
(3) The maximum value of the equilibrium adsorption capacity was observed for the modified samples with the specific surface of S_sp_ = 240 ± 24 m^2^/g. This value S_sp_ is provided by modification of the initial material with 1.2 wt % of sodium or with 2.6 wt % of potassium, which approximately corresponds to a similar mole concentration that is equal to 5.2⋅10^−4^ and 6.8⋅10^−4^ moles of the metal per gram of the initial Al_2_O_3_, respectively. Such content of Na and K cations is approximately (0.13–0.17) ML of the monolayer (ML) coating of active sites on the surface of a porous material and allows for increasing the sample capacity by ~40%.

## Figures and Tables

**Table 1:** Chemical composition and texture characteristics of aluminium oxide samples.

Sample	Composition, Mass %	S_sp_, m^2^/g	Total Pore Volume, cm^3^/g	Average Pore Diameter, nm
Al_2_O_3_	0.1 Na	290	0.34	4.7
Al_2_O_3_ + 1.3Li	1.3 Li	173	0.39	9.1
Al_2_O_3_ + 2.0Li	2.0 Li	127	0.39	12.3
Al_2_O_3_ + 4.1Li	4.1 Li	79	0.29	14.7
Al_2_O_3_ + 1.2Na	1.2 Na	250	0.40	6.5
Al_2_O_3_ + 2.4Na	2.4 Na	184	0.32	7.0
Al_2_O_3_ + 4.0Na	4.0 Na	98	0.19	7.7
Al_2_O_3_ + 1.6K	1.6 K	259	0.45	6.9
Al_2_O_3_ + 2.0K	2.0 K	247	0.38	6.3
Al_2_O_3_ + 4.5K	4.5 K	222	0.37	6.7
Al_2_O_3_ + 5.4K	5.4 K	169	0.31	7.7

**Table 2:** Kinetic characteristics of the adsorbents under study (parameters of Glueckauf equation (1)).

№ of Sample	a^*^, g_H2O_/g_ads_	β, min^−1^	R_0_^*^
Al_2_O_3_	0.25	0.0240	0.99
1.3Li	0.26	0.0158	0.91
2.0Li	0.27	0.0123	0.92
4.1Li	0.20	0.0142	0.89
1.2Na	0.35	0.0236	0.95
2.4Na	0.32	0.0216	0.95
4.0Na	0.20	0.0185	0.96
1.6K	0.34	0.0225	0.98
2.0K	0.33	0.0193	0.96
4.5K	0.33	0.0185	0.98
5.4K	0.31	0.0176	0.93

^*^ R_0_ – linear correlation coefficients.
